# Socket Preservation Using a (Dense) PTFE Barrier with or without Xenograft Material: A Randomized Clinical Trial

**DOI:** 10.3390/ma12182902

**Published:** 2019-09-08

**Authors:** Márcio de Carvalho Formiga, Ulisses Ribeiro Campos Dayube, Cristiane Kern Chiapetti, Daniela de Rossi Figueiredo, Jamil Awad Shibli

**Affiliations:** 1 Department of Periodontology and Oral Implantology, Dental Research Division, University of Guarulhos, 07023-040 Guarulhos, SP, Brazil (U.R.X.D.) (J.A.S.); 2 Department of Periodontology and Oral Implantology, Unisociesc Florianópolis, 600 Florianópolis, SC, Brazil; 3 Department of Public Health, University of South Santa Catarina-Unisul, 88137-270 Palhoça, SC, Brazil (C.K.C.) (D.d.R.F.)

**Keywords:** biomaterials, guided bone regeneration, socket preservation

## Abstract

When alveolar preservation procedures are not performed after tooth extraction, aesthetic and functional impairment could occur. Guided bone regeneration using polytetrafluoroethylene (PTFE) membranes has proven to be a simple alternative treatment that results in good maintenance of the alveolar bone for mediate/late implant placement. Therefore, this study compared the effect of alveolar preservation with the use of dense PTFE membranes, with and without xenograft material by Computerized tomography-based body composition (CTBC) analysis, after four months of the socket preservation procedure. A total of 29 teeth indicated for extraction. In the test group, the sockets were filled with bone graft biomaterial and subsequently coated with a dense PTFE membrane. In the control group, the sockets were filled with the blood clots and subsequently coated with a dense PTFE membrane. The results we found on the changes of the bone width and height after the procedures were: buccal plate: control group 0.46 mm, test group 0.91 mm; alveolar height: control group −0.41 mm, test group 0.35 mm; cervical third: control group −0.89 mm, test group −0.11 mm; middle third: control group −0.64, test group −0.50; and apical third: control group 0.09 mm, test group −0.14 mm. The use of a xenograft in conjunction with d-PTFE membranes proved to be superior to the use of the same membrane and blood clot only in regions of the crest, middle third, and alveolar height.

## 1. Introduction

Socket preservation after tooth extraction is a constant challenge for clinicians, given the importance of maintaining sufficient bone height and thickness to assist in oral rehabilitation, with or without implants. The natural bone remodeling that occurs after an extraction leads to cosmetic and functional defects, and these defects can be so severe that the placement of implants or conventional dentures can be difficult, or even impossible, without the use of some type of grafting procedure [1,2]. This socket resorption refers to the remodeling that occurs after tooth extraction, and it may result in up to 50% bone resorption, with the magnitude of horizontal resorption usually being more pronounced than the vertical resorption [3]. Socket preservation by any procedure, performed at the time of extraction for the purpose of minimizing resorption of the bone crest and buccal plate and maximizing bone formation in the alveoli, is very important. This principle, called osteopromotion, can be very successful irrespective of the cause of tooth loss [4]. On the other hand, the principles of osteoconductivity provide the space and framework for cell substrate and biochemical events to enable bone formation to occur [5].

The need for reconstruction of resorbed alveolar bone has led to a search to improve the techniques for socket preservation and to advance biomaterial studies that could replace or improve grafting procedures. Bone grafts are obtained from different sources: autologous (from the individual themselves), allogeneic (from the same species), xenogenic (from a different species), or alloplastic (synthetic) [6]. Due to the necessity to decrease the morbidity involved in removing an autogenous bone graft from a second surgical site, xenogenic, and alloplastic grafts have gained preference in guided bone regeneration procedures [7].

Sealing the post-extraction sockets with membranes protects the grafting material in the cavity and preserves high-quality soft tissue in the region. Furthermore, an appropriate space is created, where the biological potential can be expanded to assist regeneration as desired. Non-absorbable membranes clog the rapid growth of gingival epithelial cells and simultaneously create and help maintain a space where other bone cells can repopulate the area of the socket, creating conditions for new bone formation [8]. Preservation of the post-extraction alveoli plays a vital role in deciding on options for replacing missing teeth, helping to create healthy conditions so that the patient can obtain a better treatment [9].

Based on these assumptions, this study aimed to compare clinical and tomographic findings on socket preservation, four months after the patients underwent regeneration surgery. The procedures used were guided bone regeneration with a xenogenic bone graft and a dense polytetrafluoroethylene (d-PTFE) membrane barrier, compared to allowing the natural clot to fill the socket and then sealing it with the same barrier d-PTFE.

## 2. Materials and Methods

### 2.1. Study Population

This randomized clinical study was conducted with the approval of the Ethics Committee of the Dental School of South of Santa Catarina (Unisul), identified by the presentation of the Certificate of Ethical Appreciation (CAAE) Number 78141617.8.0000.5369. It was designed in concordance with the CONSORT guidelines (www.consort-statement.org) and was conducted in compliance with the principles outlined in the Declaration of Helsinki relating to clinical research involving human beings, as revised in 2013. The study was registered at www.ensaiosclinicos.gov.br with the UTN U1111-1232-1998.

The sample consisted of patients who attended the Unisul School of Dentistry undergraduate clinic in the period August to November 2017, who had teeth with indication for extraction, with extensive cavities, root fracture or vertical periodontal defects.

The patients selected were contacted and asked to sign the term of free and informed consent and a letter of consent, authorizing the collection of clinical data, allowing the interventions required for the research, use of CT data, photographs and their case histories.

### 2.2. Inclusion and Exclusion Criteria

The study included patients over 18 years of age, with no systemic diseases, good oral hygiene and teeth in need of extraction in regions where the use of a temporary prosthesis or mucosal support was not necessary.

**Figure 1:**
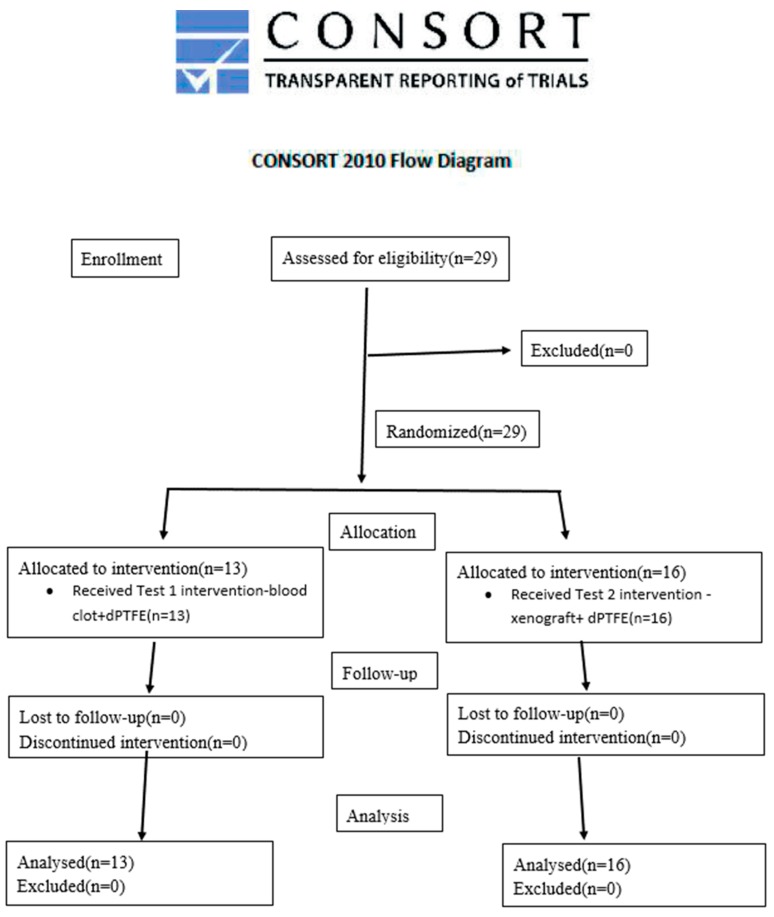
Consolidated Standards of Reporting Trials CONSORT Flow diagram.

Smokers, decompensated diabetics, pregnant women, patients with Stage III or IV periodontal disease Grade C [10] were excluded from the study. Periodontal treatment, such as scaling, root planning and eliminating active cavities, was performed to ensure the patients had an adequate oral status before inclusion in the study. Patients who attended the appointments with visible biofilm accumulation received another plaque control treatment and more oral health orientations and were instructed to return in fewer period. The Consolidated Standards of Reporting Trials (CONSORT) flow diagram inherent to the present clinical trial can be seen in Figure 1.

The ideal sample size to assure adequate power for this RCT was calculated considering differences of at least 0.5 mm in buccal bone height between groups and assuming a standard deviation of 0.4 mm previously reported^12^. Based on these calculations, it was defined that 11 teeth per group were necessary to provide an 80% power with an α of 0.05. Considering an attrition of about 20%, it has been established that at least 13 teeth should be included in each treatment group

### 2.3. Pre-Surgical Procedures

The procedures for dental extraction and the insertion of dental biomaterials follow strict aseptic conditions, according to the protocol described by the manufacturer of the biomaterials. The selected patients received a preoperative dose of oral antibiotic (2 g of amoxicillin 500 mg) 1 h before surgery, and a mouthwash containing 0.12% chlorhexidine (15 mL) was used for rinsing for about 1 min, immediately prior to the procedure. The perioral region was cleaned with antiseptic 2% chlorhexidine, and then anesthesia was performed using 4% articaine with epinephrine 1:100,000. The surgeries were performed by two surgeons with extensive experience in implantology and periodontal surgery (MCF and URCD), following the same protocol. The extraction procedures performed with the use of delicate instruments, including periotomes, in an endeavor to promote the minimal possible trauma to the tissues surrounding the tooth being removed. An intrasulcular incision was made with a 15C blade, and then delicate luxation was performed using periotomes. In the bi- and tri-rooted teeth, dental sections were performed using a high-speed drill (701), before dislocating and removing the roots. Special attention was paid to avoid damaging the soft tissues. After extraction, the socket walls were curetted to remove the granulation tissue and the remains of the periodontal ligaments [11].

### 2.4. Groups

After tooth extraction, the patients were randomly assigned to the following groups by means of a coin tossed (heads or tails) by another researcher (CKC): those whose names fell on the head side were referred to the Test group 1 (TG1; PTFE membrane + blood clot) and those on the tail side, to the Test group 2 (TG2; PTFE membrane + xenogenic bone substitute biomaterial).

For the TG2, the sockets were filled with xenogenic bovine bone-graft material (Lumina Bone Porous, Criteria^®^, Sao Carlos, SP, Brazil), composed basically by inorganic particles, biocompatible, hydrophilic and with radiopacity and internal porous, osseoconductor, up to the original level of the alveolar ridge; a dense PTFE barrier (Lumina d-PTFE, Criteria^®^, Sao Carlos, SP, Brazil), a biocompatible barrier with nano-porous, projected to be left exposed to the oral cavity, was used to seal the socket, and the membrane was stabilized with a cross suture. For the TG1, the sockets were only filled by clotting that was allowed to form naturally; after this, the same d-PTFE barrier (Lumina d-PTFE, Criteria^®^, Sao Carlos, SP, Brazil) was applied, and stabilized with a cross suture.

The ideal sample size to assure adequate power for this RCT was calculated considering differences of at least 0.5 mm in buccal bone height between groups and assuming a standard deviation of 0.4 mm [12]. Based on these calculations, it was defined that 11 teeth per group were necessary to provide an 80% power with an α of 0.05. Considering an attrition of about 20%, it has been established that at least 13 teeth should be included in each treatment group.

### 2.5. Post-Surgical Procedures

**Figure 2:**
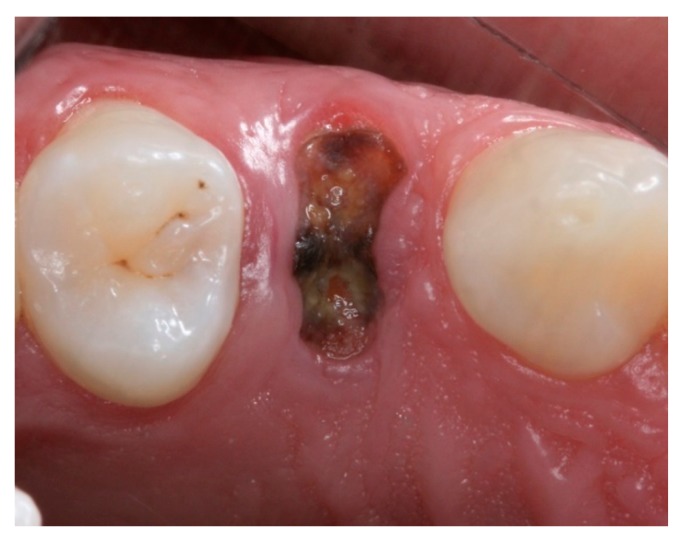
Intraoral occlusal preoperative view of tooth 14.

**Figure 3:**
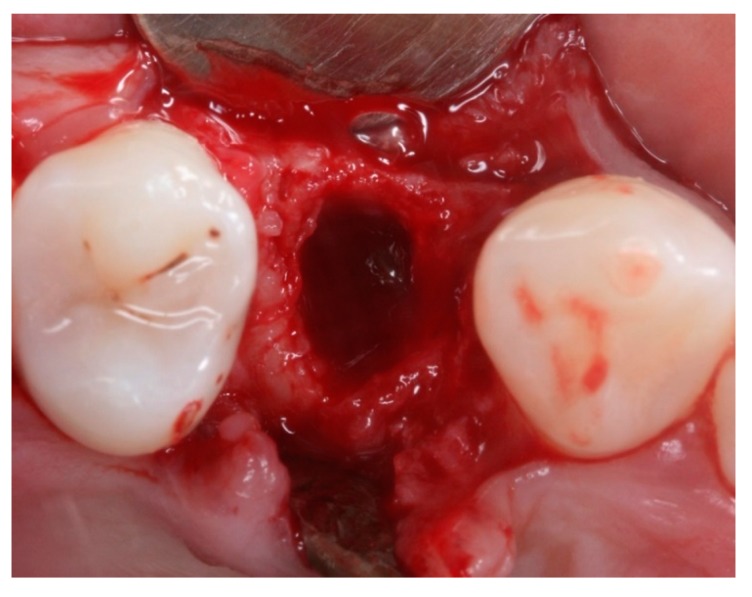
Intraoral occlusal view after extraction of tooth 14 (TG1).

**Figure 4:**
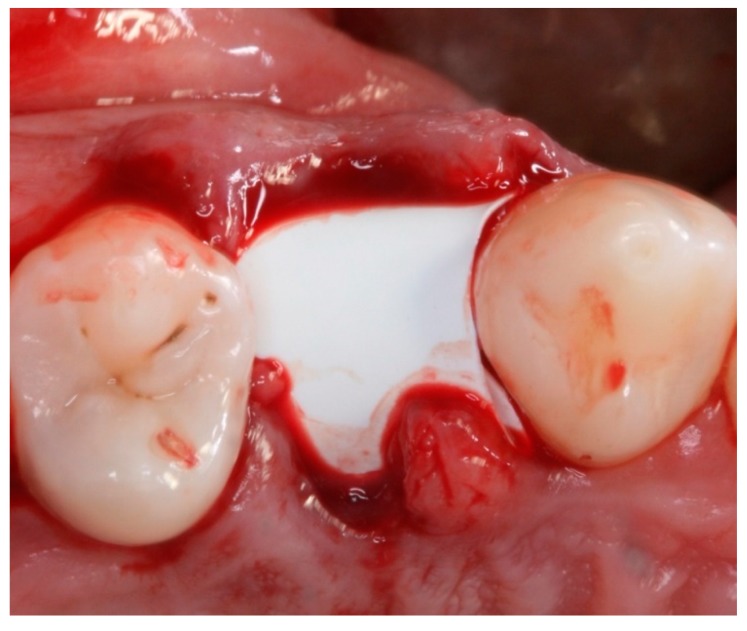
Intraoral occlusal view of the position of the a dense polytetrafluoroethylene (dPTFE) membrane (TG1).

**Figure 5:**
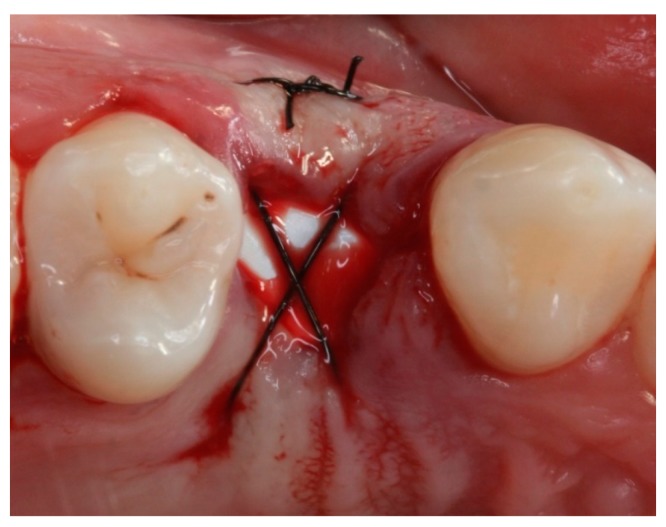
Intraoral occlusal view of the suture-stabilized membrane (TG1).

**Figure 6:**
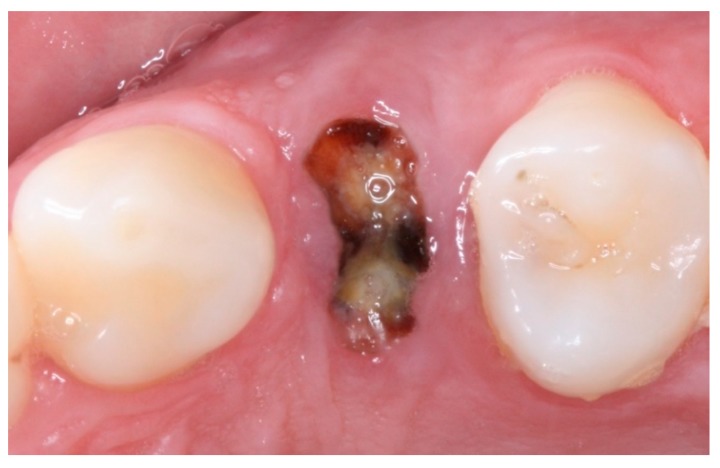
Intraoral occlusal preoperative view of the region of toot 24.

**Figure 7:**
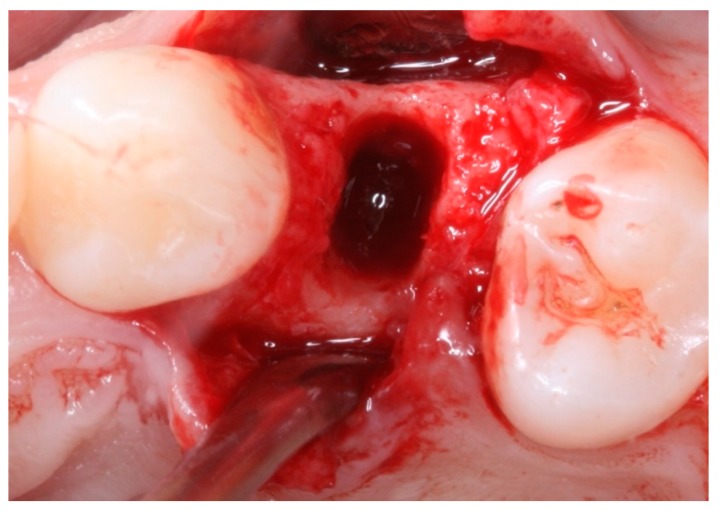
Intraoral occlusal view after extraction of tooth 24 (TG2).

**Figure 8:**
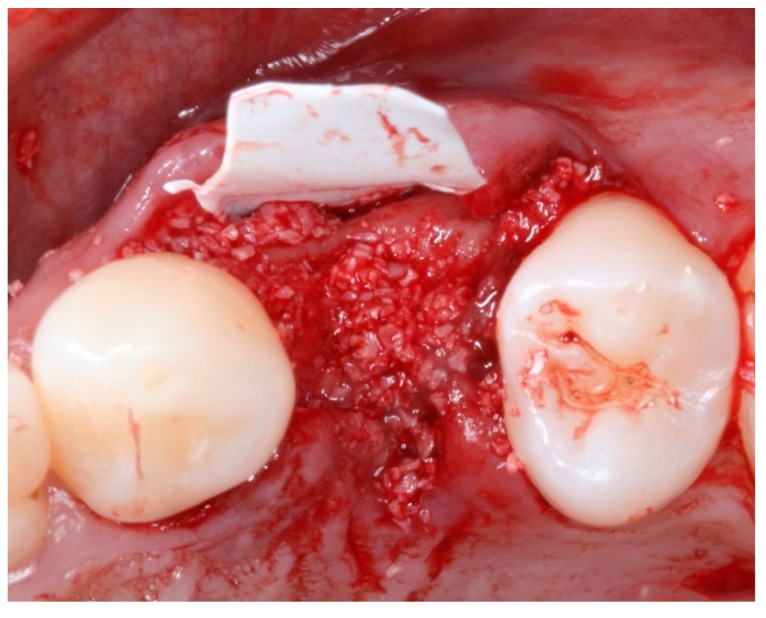
Intraoral occlusal view of the position of the d-PTFE and with the placement of the xenograft biomaterial in the socket of tooth 24 (TG2).

**Figure 9:**
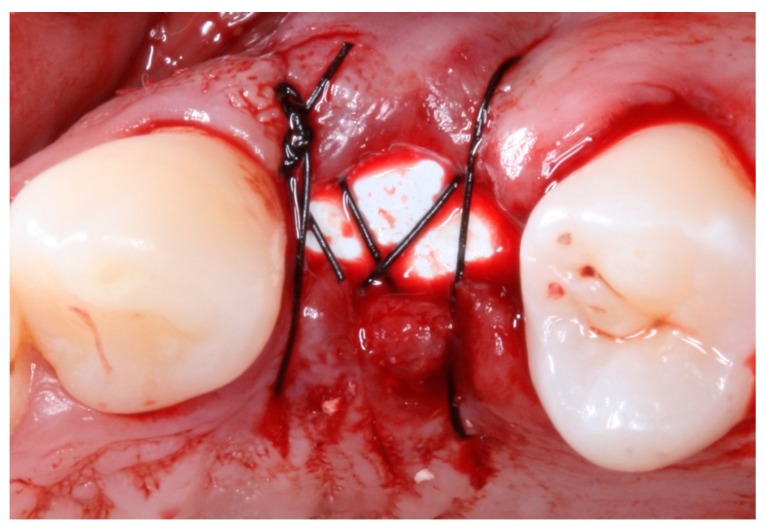
Intraoral occlusal view of the suture-stabilized membrane (TG2).

Postoperative care included continued 500 mg amoxicillin every 8 h for 7 days and paracetamol 750 mg every 6 h (for pain control), as well as use of the mouthwash with 0.12% chlorhexidine three times a day for 14 days [13]. After seven days, the sutures were removed, and at 21 days, the d-PTFE membranes were removed, without the need for local anesthesia. (Figure 2, Figure 3, Figure 4, Figure 5, Figure 6, Figure 7, Figure 8 and Figure 9). Patients were instructed to use ice bags on the surgical region within 48 h after surgeries and to eat only soft and cold food during this period.

### 2.6. Tomographic Analysis

All patients underwent cone beam CT scans at two stages in the treatment:

Prior to the surgery (extraction + PTFE membrane + biomaterial or blood clot)

In the postoperative period, after four months.

Using a software (Dental Slice, Brasilia, Brazil), two operators (MCF and URCD) made measurements of the following: (a) height of the buccal plate; (b) thickness of the socket in the cervical, middle and apical thirds; and (c) height of the socket.

**Figure 10:**
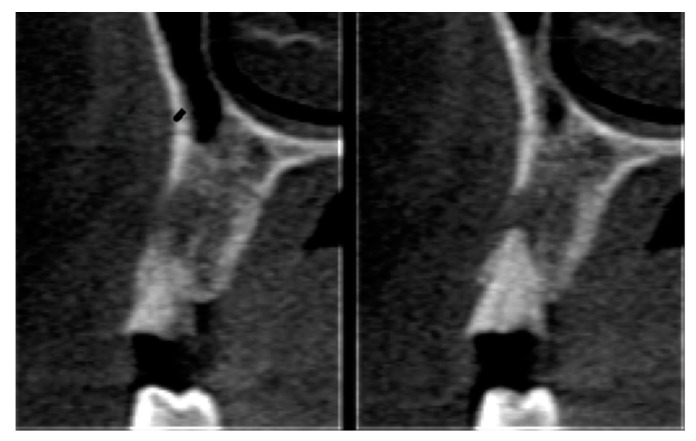
CT images of tooth 14 prior to the procedure.

**Figure 11:**
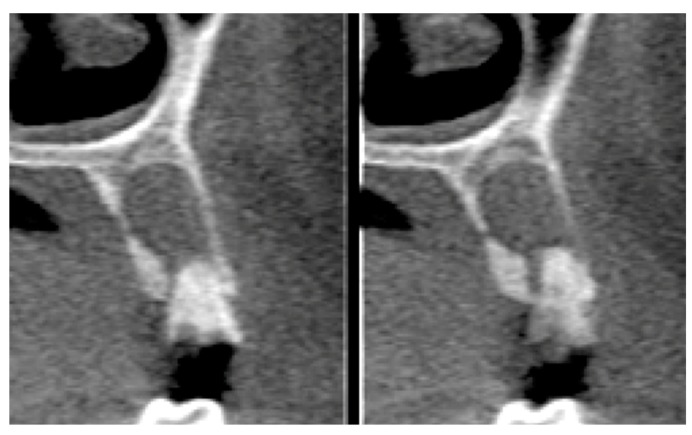
CT images of tooth 24 prior to the procedure.

**Figure 12:**
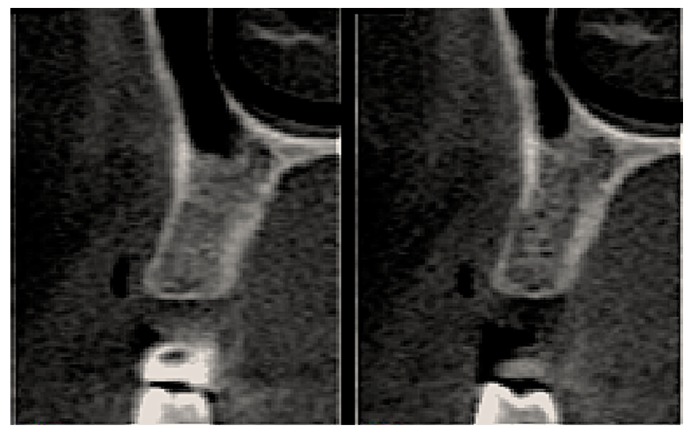
CT images of tooth 14 (TG1) 4 months after the procedure.

**Figure 13:**
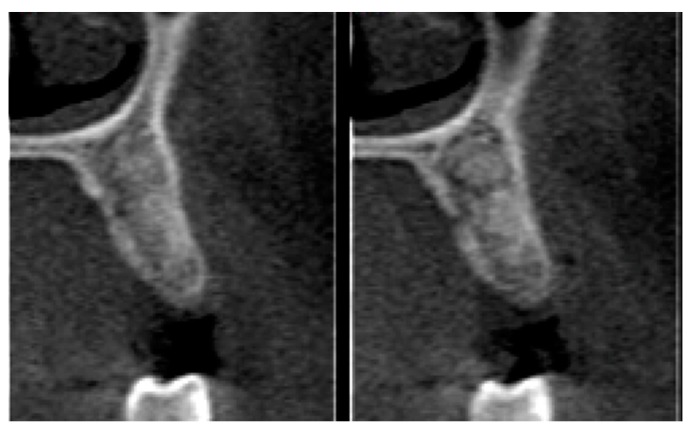
CT images of tooth 24 (TG2) 4 months after the procedure.

**Figure 14:**
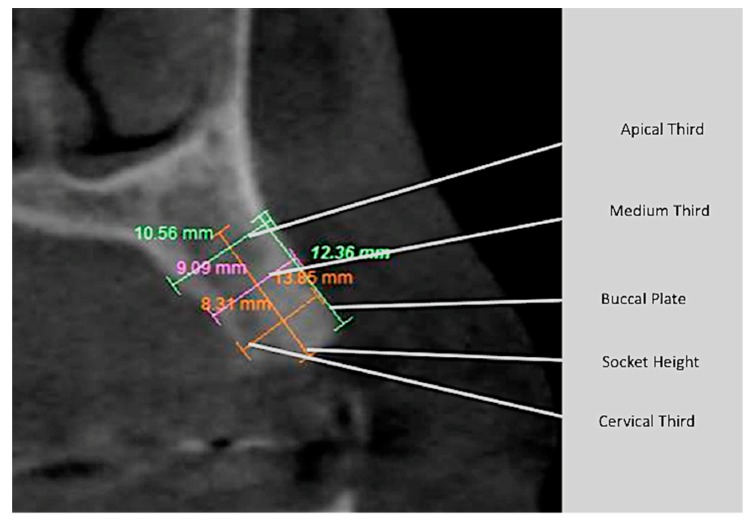
Scheme of the CT measurements, using DentalSlice software.

**Figure 15:**
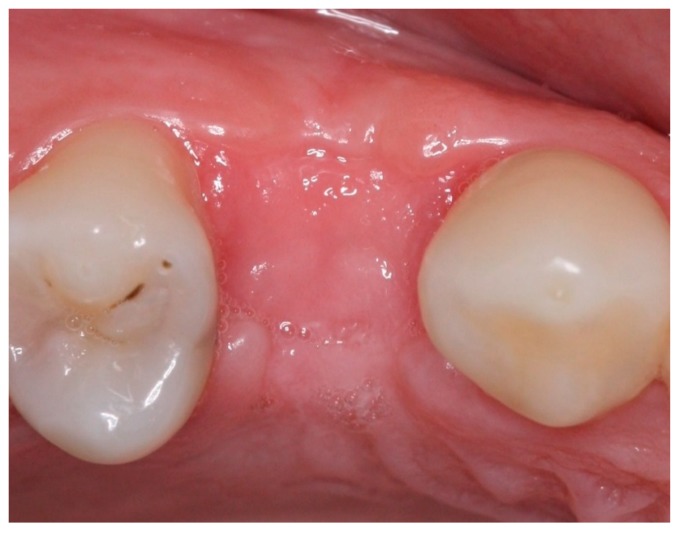
Intraoral occlusal view of TG1, four months after the procedure.

**Figure 16:**
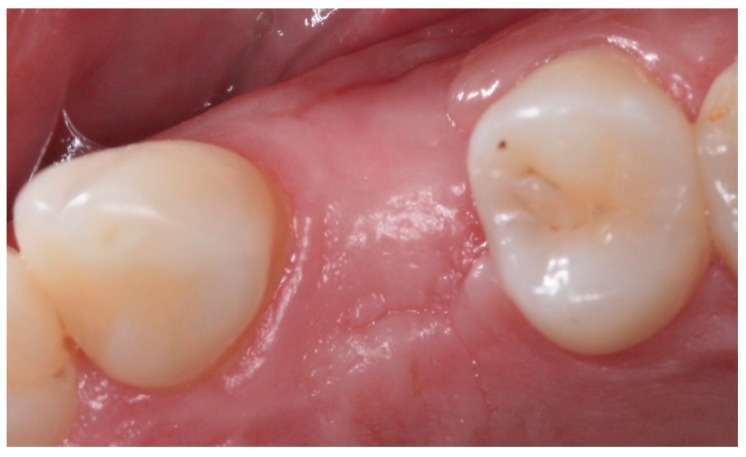
Intraoral occlusal view of TG2, four months after the procedure.

Statistical analysis was performed, using the paired-t test to determine associations, using Stata 14 (StataInc, College Station, TX, USA) (Figure 10, Figure 11, Figure 12 and Figure 13). The same operator that performed the initial measurement of a patient was responsible for the final measurement of the same patient, using the same anatomical references, as is illustrated in Figure 14. Apical third is located 2 mm near the most apical part of the socket, cervical third is located 2 mm above the most cervical part of the socket, and medium third is located in the middle of the socket, dividing it in two equal parts. Figure 15 and Figure 16 illustrate TG1 and TG2, respectively, clinical aspects after four months of healing time.

After the application of the inclusion criteria, the study included 25 patients (15 male and 10 female, mean age of 34.8 years, no smokers, 11 with periodontal disease history, but on treatment) with a total of 29 dental elements, 12 on the mandible and 17 on the upper maxilla. The TG1 comprised 13 elements, the TG2 16, all with indicated extractions, who completed the entire study. In total, two upper lateral incisors, four upper canines, four upper first molars, eight upper pre-molars, and eight lower pre-molars were included. Just one contiguous case on each group (upper lateral and canine) was included. All membranes remained in place for a period of 21 to 28 days before their removal.

Table 1 shows the results of the dimensional changes of the TG1 measurements for each parameter evaluated.

Table 2 lists the results of the measurements of dimensional change of the TG2 for each parameter evaluated.

Table 3 shows the results from comparing the means of gain or loss (negative) (in mm) between the TG1 and TG2 for each parameter evaluated. Where *p* = 0.05, a statistically significant difference existed between the groups.

## 3. Discussion

With the aid of membranes, sockets can be covered to prevent the entry of soft tissue, thus promoting maximum bone healing. Two types of membrane are used—namely, resorbable and non-resorbable [14]. Some authors have advocated that resorbable membranes do not have the capacity to maintain the spaces as well as non-resorbable PTFE membranes, and they should therefore be associated with bone grafting when there is a need to increase the bone volume of the ridge [15].

One significant clinical difference in the use of membranes with d-PTFE and those with expanded PTFE appears in the act of removal. In the case of expanded PTFE membranes, the pores allow fiber penetration, which requires dissection of the surrounding tissue at the time of removal, whilst d-PTFE membrane removal is simple due to the absence of internal tissue integration into the structure [16]. Another important characteristic of the dense PTFE membranes is the capacity of cellular occlusion, which allows epithelial and bacterial cells exclusion from the healing sites, therefore improving bone regeneration inside the dental sockets, and providing better results with respect to ridge preservation [17,18]. With this in mind, we chose to use d-PTFE in this clinical study.

Some authors have suggested filling the alveolus with only the clot, followed by coating with a membrane of d-PTFE, for the purpose of recovering the alveolar ridge/bone architecture and promote bone formation. Four months after surgery, bone regeneration and maintenance of the alveolar architecture was observed, which allowed placement of a dental implant [19]. This result was also observed in our study, using d-PTFE membranes, both with and without the use of bovine bone substitute.

We now know that particulate bovine bone has high porosity, which allows the proliferation of blood vessels and migration of bone cells. As it resembles human bone, it allows osteopromotion to occur more naturally [20]. Other studies have compared socket preservation with biomaterials versus clots after extraction and concluded that in all cases the patients showed significantly better maintenance of crest width by using socket preservation with bone grafts in comparison with healing the socket with blood clots [21]. The same condition was evident in our study, where we obtained statistically significant differences between the study groups in the important parameters for planning implant placement; that is, the height of the socket and the middle and cervical thirds—crucial regions for better aesthetics in peri-implant tissues.

Aranha and Braga, 2011 [22], compared the success of dental implants in certain grafted biomaterials. Using the same xenograft material evaluated in our study, they found that it met the function of supporting bone repair for further implants, according to the usual guided bone regeneration protocol. Noia et al., 2015 [23], considered it important to fill the vestibular gap with osteoconductive xenograft material, as filling this with bone biomaterial was an essential factor in obtaining stability of the results over the years in immediate dental implant placement. Another study assumed that this material was a bovine bone substitute and reported that the material had an osteoconductive function that contributed to bone tissue formation and the growth of blood vessels inside defects and particles [24]. Although there was no difference between the groups in terms of bone gain on the buccal plate, in three of the other four parameters evaluated, better results were achieved using xenograft material for bone gain inside the socket.

Another study evaluated post-extraction sockets in the maxilla, in which patients were divided into two groups: a TG with xenograft material (Bioss^®^) (Geistlisch, Wolhusen, Switzerland) fillings and the CG that used the traditional procedure. A first CT scan was made 30 days after surgery, in which no significant changes were seen. A second CT scan was performed at 90 days, in which significant height loss was observed in the CG compared with the TG. The authors concluded that the use of biomaterials in the alveoli appeared to be slightly significant, especially when they were used in aesthetic areas [25]. Furthermore, in 2009, Araujo and Lindhe [26] compared post-extraction sockets divided into two groups: a TG with the alveolus filled with xenograft material, and a CG with only the blood clot. After six months, histometric analyses were performed in the thirds of the alveolar sockets. In the apical region, the results were similar between the groups, but in the coronal region, the CG had a much higher resorption rate than the TG. Since it has previously been clearly shown that if nothing is done at the time of tooth extraction, the patient would be subject to an average absorption of 50% of the socket volume [3], we considered that the procedure used for the TG1 in our study would be of some benefit to the patient. Therefore, we chose to use natural clotting, but covered the alveoli with a d-PTFE membrane, the benefits of which have been well documented, when used appropriately in socket preservation procedures [9,27,28,29].

Fotek et al., in 2009 [27] performed histomorphometric analysis to find more vital bone on the dense PTFE group than on the AlloDerm group (allogenic material), but without statistical significance. Ronda et al., in 2014 [16], also showed no difference between the quality of the newly bone formed under either a dense or expanded PTFE barrier for guided bone regeneration. But Iasella et al., in 2003 [21], found that on the group left to heal with only blood clot the total amount of vital bone was higher than the one with allograft, although this group had a better result on the volume after ridge preservation. These findings can be explained by the presence of a bone substitute, which stay on the area for a longer time till resorption or incorporation, if happened any of them. But on sites with only blood clot, only natural new bone is expected to be formed, but with a smaller volume due to its contraction. The same is expected on our study, even that histomorphometric analysis wasn’t an outcome.

As a limitation of our study, we chose to include teeth with different root anatomies, from lateral incisors to first molars, in order to test the viability of the biomaterials on all usual situations, once in our private practice, it is not common to use different materials according to different tooth positions on the mouth or arch. Because of that, we didn’t distinguish between the results of mandible from maxillae, even though it is known that they have different patterns of resorption, and this may generate a risk of bias with respect to the results.

## 4. Conclusions

Within the limitations of this study, we concluded that the use of d-PTFE membranes was effective in alveolar preservation, with or without the adjunct use of particulate bovine bone grafts. The use of particulate bovine bone substitutes, in conjunction with the use of d-PTFE membranes, resulted in less bone loss in the middle and cervical thirds of the sockets. In addition, better results were achieved in socket height maintenance when compared with the blood clot and dense-PTFE membrane alone.

We recommend further studies with longer follow-ups in order to confirm the stability of these results.

## Figures and Tables

**Table 1:** Mediums and standard deviations (in mm) of the evaluated parameters in the TG1, before, and four months after, the procedure (blood clot and d-PTFE membrane for socket preservation), based on CT analysis.

Parameter	Before		After	
	Mean	Standard Deviation	Mean	Standard Deviation
Buccal plate	5.55	1.73	6.01	2.33
Socket height	9.10	1.50	8.69	1.48
Cervical third	7.46	0.73	6.57	1.12
Medium third	7.80	1.00	7.15	1.10
Apical third	8.10	1.17	8.18	1.32

Paired t test. Source: Prepared by the authors (2019).

**Table 2:** Means and standard deviations (in mm) of the evaluated parameters in the TG2, before, and four months after, the procedure (particulate bovine bone and d-PTFE membrane for socket preservation), based on CT analysis.

Parameter	Before		After	
	Mean	Standard Deviation	Mean	Standard Deviation
Buccal plate	7.37	2.32	8.49	1.95
Socket height	9.96	1.36	10.32	2.07
Cervical third	7.68	0.94	7.57	0.92
Medium third	8.10	1.29	8.02	1.31
Apical third	8.78	1.26	8.64	1.46

Paired t test. Source: Prepared by the authors (2019).

**Table 3:** Differences between the means (in mm) of the gains/losses between the TG1 and TG2 for each parameter.

Parameter	Control		Test		*P* Value
	Mean	Standard Deviation	Mean	Standard Deviation	
Buccal plate	0.46	3.04	1.11	0.91	0.417
Socket height	−0.41	0.76	0.35	1.16	0.049
Cervical third	−0.89	0.71	−0.11	0.73	0.008
Medium third	−0.64	0.57	−0.50	0.32	0.002
Apical third	0.09	0.60	−0.14	0.69	0.348

* Statistical difference—paired t test. Source: Prepared by the authors (2019).
